# Anaesthesia for total hip and knee replacement: A review of patient education materials available online

**DOI:** 10.12688/f1000research.18675.1

**Published:** 2019-04-09

**Authors:** Rebecca Marshall, Eoghan Pomeroy, Catriona McKendry, Michael Gilmartin, Paula McQuail, Mark Johnson

**Affiliations:** 1 Department of Anaesthesia, University Hospital Galway, Galway, Connaght, H91 YR71, Ireland; 2 Department of Orthopaedics, Mater Misericordiae University Hospital, Dublin, Dublin, Ireland; 3 Department of Orthopaedics, St. James's Hospital, Dublin, Dublin, Ireland; 4 School of Medicine, University College Dublin, Dublin, Ireland; 5 Department of Anaesthesia, Fiona Stanley Hospital, Murdoch, Australia

**Keywords:** Anaesthesia, Quality, Readability, Total knee replacement, Total hip replacement, Internet, Patient information.

## Abstract

**Background:** Patients frequently consult the internet for health information. Our aim was to perform an Internet-based readability and quality control study using recognised quality scoring systems to assess the patient information available online relating to anaesthesia for total hip and knee replacement surgery.

**Methods:** Online patient information relating to anaesthesia for total hip and knee replacement was identified using Google, Bing and Yahoo with search terms *‘hip replacement anaesthetic’*, *‘knee replacement anaesthetic*.’ Readability was assessed using Flesch Reading Ease (FRE), Flesch-Kincaid grade level (FKGL) and Gunning Fog Index (GFI). Quality was assessed using DISCERN instrument, Health On the Net Foundation seal, and Information Standard mark.

**Results:** 32 websites were analysed. 25% were HONcode certified, 15.6% had the Information Standard. Mean FRE was 55.2±12.8. Mean FKGL was 8.6±1.9. Six websites (18.8%) had the recommended 6 ^th^-grade readability level. Mean of 10.4±2.6 years of formal education was required to read the websites. Websites with Information Standard were easier to read: FKGL (6.2 vs. 9, *P < 0.001*), GFI (8.8 vs. 10.7, *P = 0.04*), FRE score (64.2 vs. 9, *P = 0.02*). Mean DISCERN score was low: 40.3 ± 13.

**Conclusions:** Overall, most websites were poor quality with reading levels too high for the target audience. Information Standard NHS quality mark was associated with improved readability, however along with HONcode were not found to have a statistically significant correlation with quality.  Based on this study, we would encourage healthcare professionals to be judicious in the websites they recommend to patients, and to consider both the readability and quality of the information provided.

## Introduction

Total hip replacement (THR) and total knee replacement (TKR) are proven interventions for patients with advanced arthritis, and are among the most common elective surgical procedures carried out in the UK and Ireland ^1^. National Joint Registry data for both the UK and Ireland reveals that close to 200,000 total hip and knee replacements are performed each year ^2, 3^. Demand for both THR and TKR is set to increase dramatically in the coming decades due to changing demographics and an ageing population, with studies suggesting the demand for TKR in the United States will grow by 673% by 2030 ^4– 6^. Anaesthesia can play a significant role in reducing perioperative morbidity, and in an increasingly complex patient population it is important that patients are given accurate and up to date information about the various anaesthetic techniques used ^7^.

Internet use is increasing worldwide, with 85% of adults in the United States using the Internet ^8^. In the UK, the Oxford Internet Survey group stated that in 2013, 78% of people used the Internet and, of these, up to 71% sought health related information ^9^. Patient education materials (PEM) can be beneficial for assisting patients in the informed consent procedure, by explaining indications, risks, benefits and alternatives ^10^. A recent online poll revealed that up to 90% of patients who access the Internet for their health information believe it to be accurate, and over 60% reported that it impacted their medical decision making ^11^. However, the Internet is a completely unregulated source susceptible to provider bias and has the capacity to negatively influence consumer health outcomes ^12^. Implementing and enforcing standards is very difficult, and health information available has been shown to be of poor quality and largely unreliable ^8, 11, 13, 14^. It is important for doctors to be aware of the information available to patients on the Internet and to understand confusion surrounding such information. As well as supplying high quality accurate health information, the readability of the website must be suitable for the target audience. Several medical organisations , including the National Institute of Health (NIH), and the American Medical Association (AMA) recommend that all PEM should be written at or below sixth grade level (reading age 11-12 years) in order to be effectively understood by the general public ^15^. However many previous studies have shown that a significant proportion of health information websites are written above this recommended level, suggesting that it may be beyond the comprehension of a substantial proportion of the patient population accessing it 16– 18.

Over the past number of years there has been several studies assessing both the quality and readability of PEM available on the Internet across all medical specialties, including general and regional anaesthesia for labour and pain procedures ^19– 22^. We also already know that orthopaedic patients research their conditions extensively online ^11, 13, 23, 24^, but to our knowledge none of these studies have looked at the availability of high quality health information relating to anaesthesia for common surgical procedures. Therefore, our aim was to assess the readability and quality of patient information available on the Internet relating to anaesthesia for both TKR and THR. 

## Methods

### Ethics

According to the policy activities that constitute research at the institute in question, this work met criteria exempt from ethics review.

### Search engines

On 26/09/2017 the search terms ‘hip replacement anaesthetic’ and ‘knee replacement anaesthetic’ were entered into the top three most commonly used search engines for 2017: Google, Bing and Yahoo. Most Internet users do not go beyond the first three pages of returned searches ^22^, so we only included those websites in the analysis; 27 sites for each of the above terms on both Google and Bing (9 websites per page) and 30 for each on Yahoo (10 websites per page), giving a total of 168. Websites were then excluded from further analysis if they were not PEM, if they were written in a language other than English, if they were inaccessible, or in a non-written format, i.e. video. Duplicate websites were also excluded. In total 32 unique websites were identified for examination, as shown in Table 1.

**Table 1.** List of analysed websites. Websites listed by search engine, authorship group and the presence or absence of the HONcode and/or Information Standard NHS quality marks. NPO, Not for profit organisations.

Search engine	Authorship	Site	Quality mark
**GOOGLE**			
**1**	Academic	https://patient.info/health/anaesthetic-choices-for-hip-or-knee-replacement	Information standard
**2**	Academic	https://www.anaesthesia.ie/attachments/article/311/Anaesthetic%20Choices%20for%20Hip%20or%20Knee%20Replacement.pdf	-
**3**	Academic	http://orthoinfo.aaos.org/topic.cfm?topic=a00372	-
**4**	Government/ NPO	http://www.nhs.uk/Conditions/Hip-replacement/Pages/How-it-is-performed.aspx	Information standard
**5**	Commercial	https://www.allinahealth.org/Health-Conditions-and-Treatments/Health-library/Patient-education/Total-Hip-Replacement/Surgery-and-beyond/anesthesia/	HONcode
**6**	Social/ discussion	https://bonesmart.org/forum/threads/hip-replacement-under-epidural-injection.9633/	-
**7**	Media-related	http://www.dailymail.co.uk/health/article-2352907/Rather-general-anaesthetic-I-watched-TV-surgeons-sawed-bone-new-hip.html	-
**8**	Commercial/ Physician	http://holycrossleonecenter.com/blog/regional-or-general-anesthesia-which-is-preferred-for-hip-or-knee-replacement-surgery/	-
**9**	Academic	http://dudleygroup.nhs.uk/wp-content/uploads/2014/03/SpinalAnaesthesiaForHipAndKneeJointReplacementSurgery.pdf	-
**10**	Commercial/ Physician	http://www.mcminncentre.co.uk/anaesthetic-procedure.html	-
**11**	Academic	http://www.wsh.nhs.uk/CMS-Documents/Patient-leaflets/AnaestheticDepartment/5546-4AnaestheticChoicesForHipOrKneeReplacement.pdf	-
**12**	Commercial	https://thrive.kaiserpermanente.org/care-near-you/northern-california/sanjose/wp-content/uploads/sites/7/2015/10/Anesthesia-for-Total- Hip-Knee-Replacement_tcm28-695230.pdf	-
**13**	Commercial	https://www.allinahealth.org/Health-Conditions-and-Treatments/Health-library/Patient-education/Total-knee-Replacement/Surgery-and- and-beyond/anesthesia/	HONcode
**14**	Academic	https://uihc.org/health-library/spinal-anesthesia-used-during-total-knee-replacement-surgery	-
**15**	Physician	https://www.mykneeguide.com/the-hospital/anesthesia	HONcode
**16**	Government/ NPO	http://www.wsh.nhs.uk/CMS-Documents/Patient-leaflets/AnaestheticDepartment/6067-1-Knee-Replacement---Anaesthesia-for-knee- replacement-surgery-(MOJO-Technique).pdf	-
**17**	Social/ discussion	https://bonesmart.org/forum/threads/general-or-spinal-block-for-anesthesia.7221/	-
**18**	Academic	http://www.cochrane.org/CD010278/ANAESTH_pain-control-using-local-anaesthetics-improve-surgical-results-after-shoulder-hip-and-knee	-
**BING**			
**19**	Government/ NPO	https://www.arthritisresearchuk.org/arthritis-information/surgery/hip-replacement-surgery/how-should-i-prepare.aspx	-
**20**	Physician	http://arthritis.emedtv.com/hip-replacement/total-hip-replacement-anesthesia.html	HONcoce
**21**	Social/ discussion	https://patient.info/forums/discuss/anaesthetic-options-534515	Information standard
**22**	Commercial/ Physician	https://www.bupa.co.uk/health-information/directory/h/hip-replacement	HONcode Information standard
**23**	Commercial/ Physician	https://www.materprivate.ie/dublin/centre-services/all-services/total-hip-replacement/Total_Hip_Replacement.pdf	-
**24**	Government/ NPO	https://www.hse.ie/eng/health/az/H/Hip-replacement/	-
**25**	Social/ discussion	https://healthunlocked.com/blf/posts/130119836/copd-and-general-anaesthetics	-
**26**	Commercial/ Physician	https://www.sportssurgeryclinic.com/services/orthopaedics/total-knee-replacement/	-
**27**	Commercial/ Physician	http://www.essexkneesurgery.co.uk/anaesthesia/	-
**28**	Commercial	https://www.bupa.co.uk/health-information/directory/k/knee-replacement	HONcode Information standard
**29**	Physician	https://www.verywell.com/best-anesthesia-for-joint-replacement-surgery-2549546	HONcode
**30**	Social/ discussion	https://treato.com/Knee+Replacement,Spinal+Anesthesia/?a=s	-
**31**	Physician	http://arthritis.emedtv.com/knee-replacement/total-knee-replacement-anesthesia.html	HONcode
**YAHOO**			
**32**	Commercial/ Physician	http://www.french-orthopaedic.co.uk/pages/anaesthtic-choices-for-hip-or-knee-replacement.pdf	-

### Scoring systems

Website authorship was determined independently by close examination by the first two authors (R.M, E.P.) and each one was placed in one of the following categories: 1) physician, author or authors were individual or group physicians with no university or research group; 2) academic, author or authors were affiliated with a university or research group; 3) commercial site, author or authors were marketing a product related to the subject; 4) commercial/physician, author or authors were individual or group physicians also marketing a product related to the subject; 6) Government/Not for profit organisations (NPO), author or authors were affiliated with a government or registered charity; 7) media-related, author or authors affiliated with the media; and 7) social/discussion, to reflect the growing trend of the use of these modalities to distribute information ^12^.

### Readability

The readability of a text is determined as the education level a person completed to understand the written material, based on the US reading grade level ^10^. We assessed the readability of each website using three validated, commonly used readability assessment tools: the Flesch Reading Ease (FRE) score, the Flesch-Kincaid grade level (FKGL), and the Gunning Fog Index (GFI). The FRE score generates a score between 0–100, using the formula: 206.835 − 1.015(total words/total sentences) − 84.6(total syllables/total words). It is based on the total words, syllables and sentences in a written passage and a score <60 considers the document to be difficult to read by the general public ^8, 19^. The FKGL corresponds to the US reading grade level and is calculated using the formula: 0.39(total words/total sentences) + 11.8(total syllables/total words) − 15.59. The GFI is calculated using the formula: 0.4[(words/sentences) + 100(complex words/words), and estimates the number of years of formal education required to read a passage of text ^8^. Readability scores for all PEM websites were generated using an online readability calculator.

### Quality

The quality of each website was assessed by the two authors using the DISCERN instrument, and according to the presence or absence of the Health On the Net (HON) Foundation seal and the Information Standard mark. The DISCERN instrument is a validated rating tool of the quality of health information developed by the NHS Executive Research and Development Programme. It consists of 15 key questions plus an overall quality rating and generates a score between 80 (highest) and 16 (lowest), a lower score being reflective of a website that is of poor quality information on treatment choices ^25^. The HON Foundation criteria was developed in 1995 by a non-profit, non-governmental organisation, accredited to the Economic and Social Council of the United Nations, in an attempt to improve the quality of internet-based health information. The HON Foundation provides a code of conduct seal for websites that meet its quality and reliability standards ^8, 14^. The Information Standard quality mark was established by the UK Department of Health to help patients and the public make informed choices about their lifestyle, condition and options for treatment and care. It is a certification scheme for health and social care information, which indicates that an organisation is a reliable source of health and social care information.

### Statistical analysis

Calculations were performed using SPSS version 23 (SPSS, Inc., Armonk, NY). Mean scores and standard deviation are presented for normally distributed variables. Median values and standard deviation are presented for non-normally distributed data. One-way ANOVA/Independent samples Kruskal-Wallis Test were used as appropriate in intergroup comparisons. In comparisons between certified and noncertified groups, independent samples T test/Mann Whitney U test were applied as appropriate. Significance was set at *P < 0.05* for all studies.

## Results

**Figure 1.**
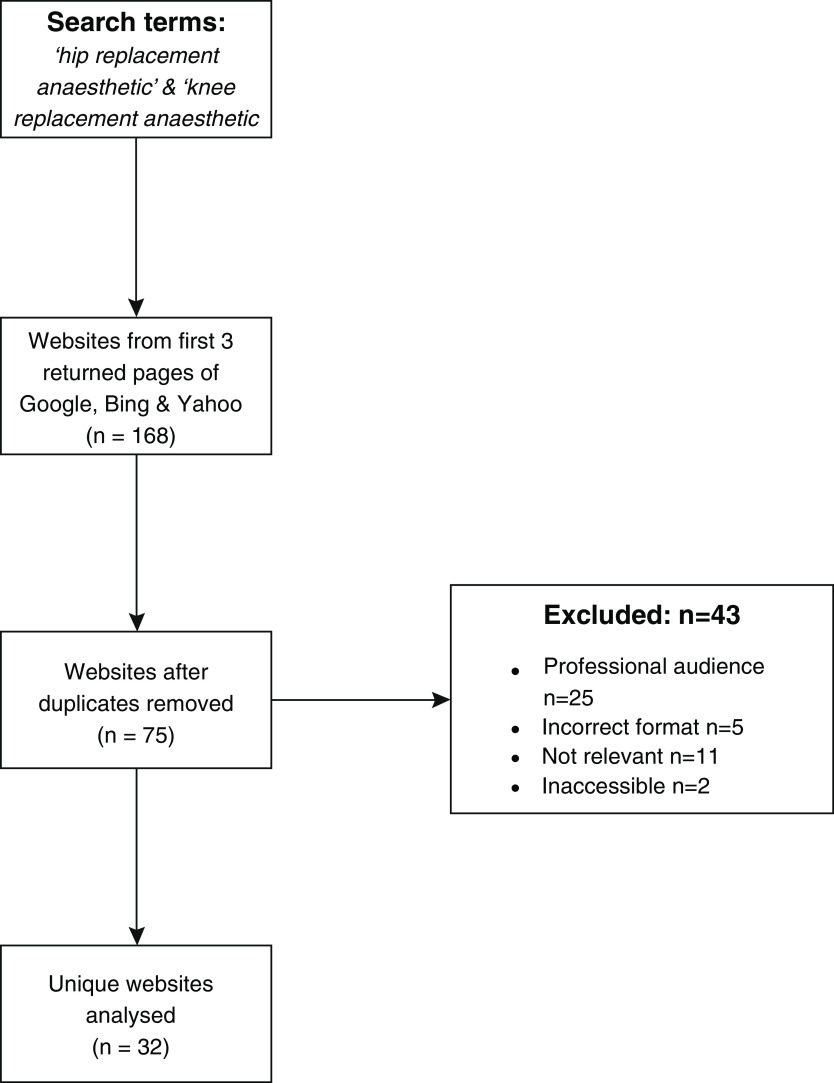
Results flow diagram of analysed websites. 27 sites for each of the above terms on both Google and Bing (9 websites per page) and 30 for each on Yahoo (10 websites per page), giving a total of 168. Websites were then excluded from further analysis if they were not PEM, if they were written in a language other than English, if they were inaccessible, or in a non-written format i.e. video. Duplicate websites were also excluded. In total 32 unique websites were analysed further.

**Figure 2.**
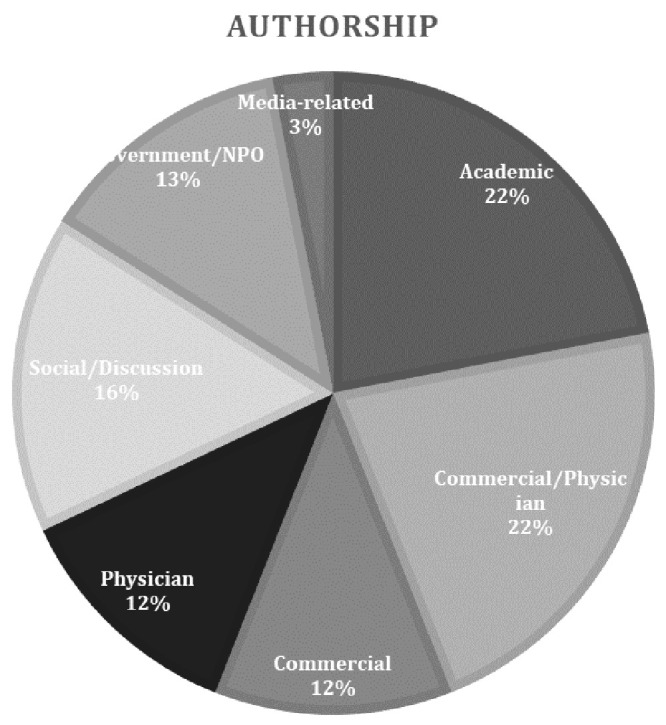
Each website was categorised according to authorship. Seven were academic, seven were commercial/physician, five were discussion/social media related, four were physician, four were Government/NPO related, four were commercial and one was media-related.

Out of the 168 initial search results, 32 were analysed further as per the previously described exclusion criteria ( Figure 1). Each website was categorised according to authorship; seven were academic, seven were commercial/physician, five were discussion/social media related, four were physician, four were Government/NPO related, four were commercial and one was media-related ( Figure 2). Only 8/32 sites (25%) were HONcode certified and 5/32 (15.6%) had the Information Standard quality mark.

### Readability

**Figure 3.**
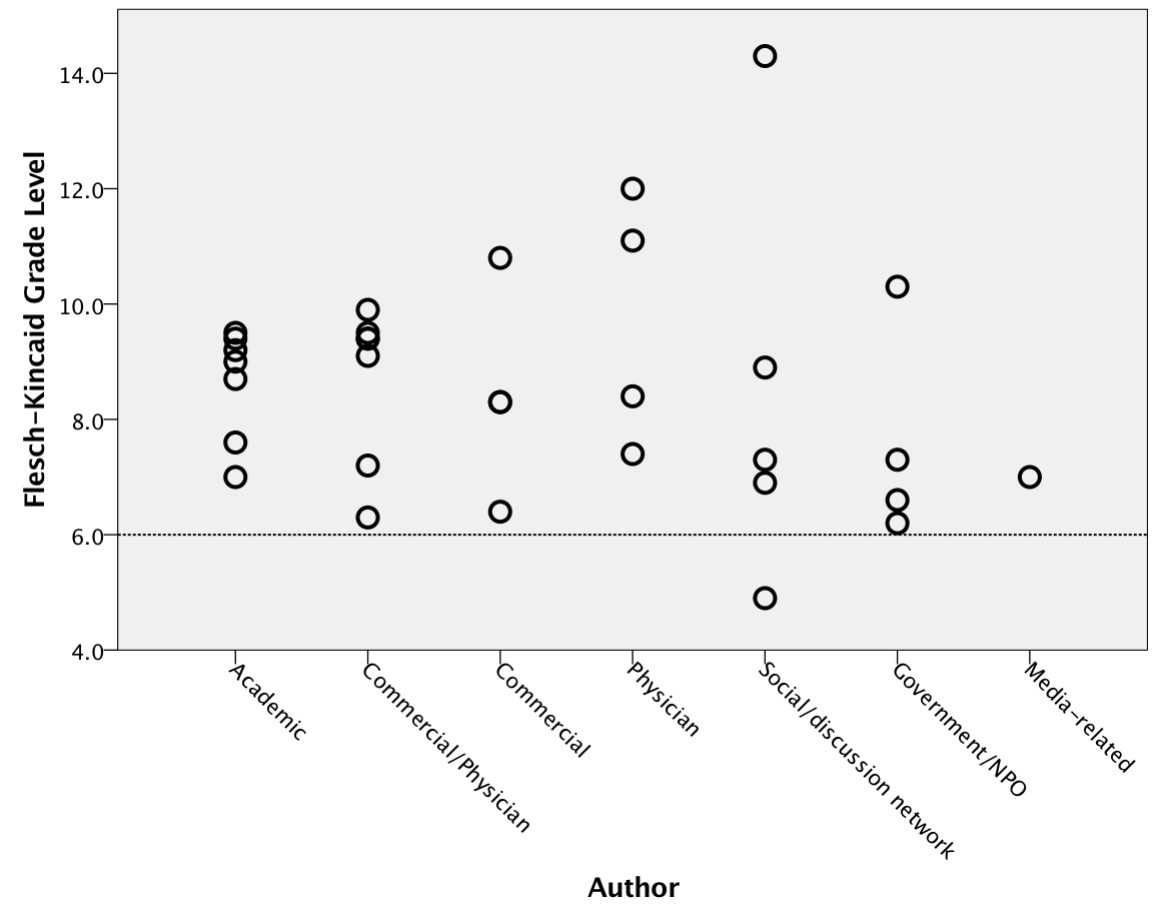
Flesch-Kincaid Grade Level by authorship. The line denotes the recommended readability level of the sixth-grade level with a minority of websites falling at or below this level.

The readability of each website was assessed using three validated commonly used readability assessment tools: the Flesch Reading Ease (FRE) score, the Flesch-Kincaid grade level (FKGL), and the Gunning Fog Index (GFI). Table 2 summarises the study’s readability and quality scores. The overall mean readability scores indicated that the websites as a group were difficult to read. The mean FRE score was 55.2 ±12.8, with social/discussion networks associated with both the minimum (3.3) and maximum (74.2) FRE scores. The mean FKGL score was 8.6 ±1.9, with only six websites (18.8%) having the recommended readability level of sixth-grade or less ( Figure 3). Overall, a mean of 10.4 years (mean GFI 10.4 ±2.6) of formal education was required to read the websites included in this study. Commercial websites had the highest mean GFI (11.7 ±2.3), while social/discussion networks had the lowest (8.3±3.9).

**Table 2.** Readability and quality values across all authorship groups. Overall results for each scoring system, presented as mean ±standard deviation for normally distributed data and median ±standard deviation for non-normally distributed data (FRE Score). FRE, Flesch Reading ease; FKGL, Flesch-Kincaid Grade Level; GFI, Gunning Fox Index; DISCERN, DISCERN instrument.

	FRE Score	FKGL	GFI	DISCERN
Total	55.2 ±12.8	8.6±1.9	10.4±2.6	40.3±13
Academic	54.8±7.1	8.6±1	10.6±2.1	49.7±11.8
Commercial/physician	55.9±8.0	8.7±1.4	10.9±1.7	38±14.3
Commercial	51.8±8.3	8.5±1.8	11.7±2.3	43±6.7
Physician	46.6±3.6	9.7±2.2	11.4±3.2	47.8±6.8
Social/discussion	70.1±30.1	8.5±3.6	8.3±3.9	24.8±3.4
Government/ Not for profit organisations	56.5±4.6	7.6±1.9	10.1±2.1	41.8±10.8
Media-related	N/A	N/A	N/A	N/A
HON-code +	51.2±8.1	8.5±2.1	10.8±2.4	45.4±6.7
HON-code -	56.4±14.1	8.6±1.9	10.3±2.7	38.5±14.2
Information Standard +	64.2±5.4	6.2±.8	8.8±1.2	42.4±12.6
Information Standard -	51.9±13	9.0±1.7	10.7±2.7	39.9±13.3

Organisations that have achieved the Information Standard quality mark were as a group easier to read. This was seen across all three readability assessment tools. Member websites had a significantly lower mean FKGL (6.2 vs. 9, *P < 0.001*) and GFI (8.8 vs. 10.7, *P = 0.04*) and a significantly higher median FRE score (64.2 vs. 9, *P = 0.02*) than non-member websites. There was no difference in FKGL (8.5 vs. 8.6, *P = 0.78*), GFI (10.8 vs. 10.3, *P = 0.92*) or FRE (51.2 vs. 56.4, *P = 0.31)* between HONcode certified and noncertified groups.

## DISCERN Instrument

**Figure 4.**
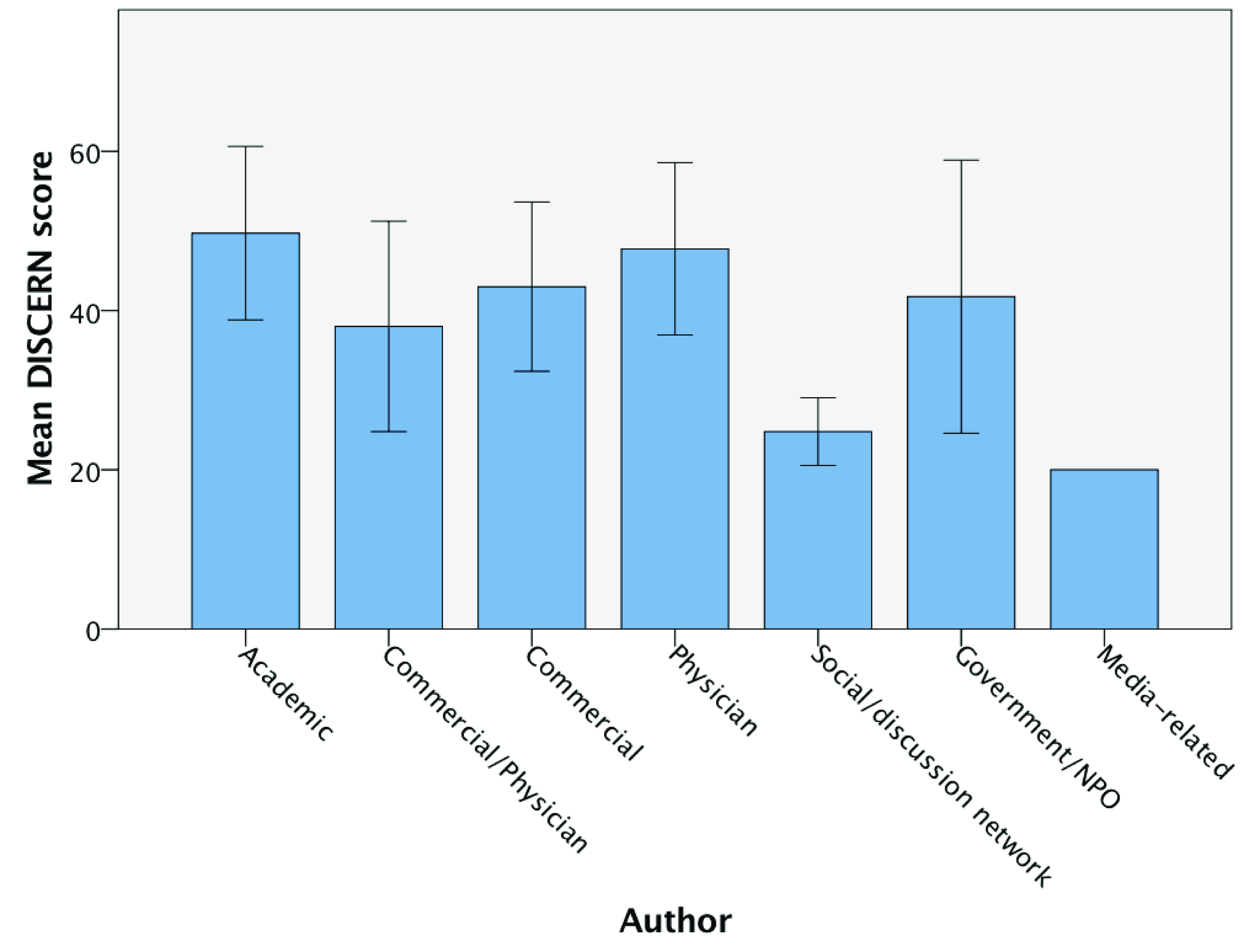
Bar chart of DISCERN scores by authorship. From highest (61) to lowest (20) mean DISCERN score. Error bars denote 95% confidence interval.

**Figure 5.**
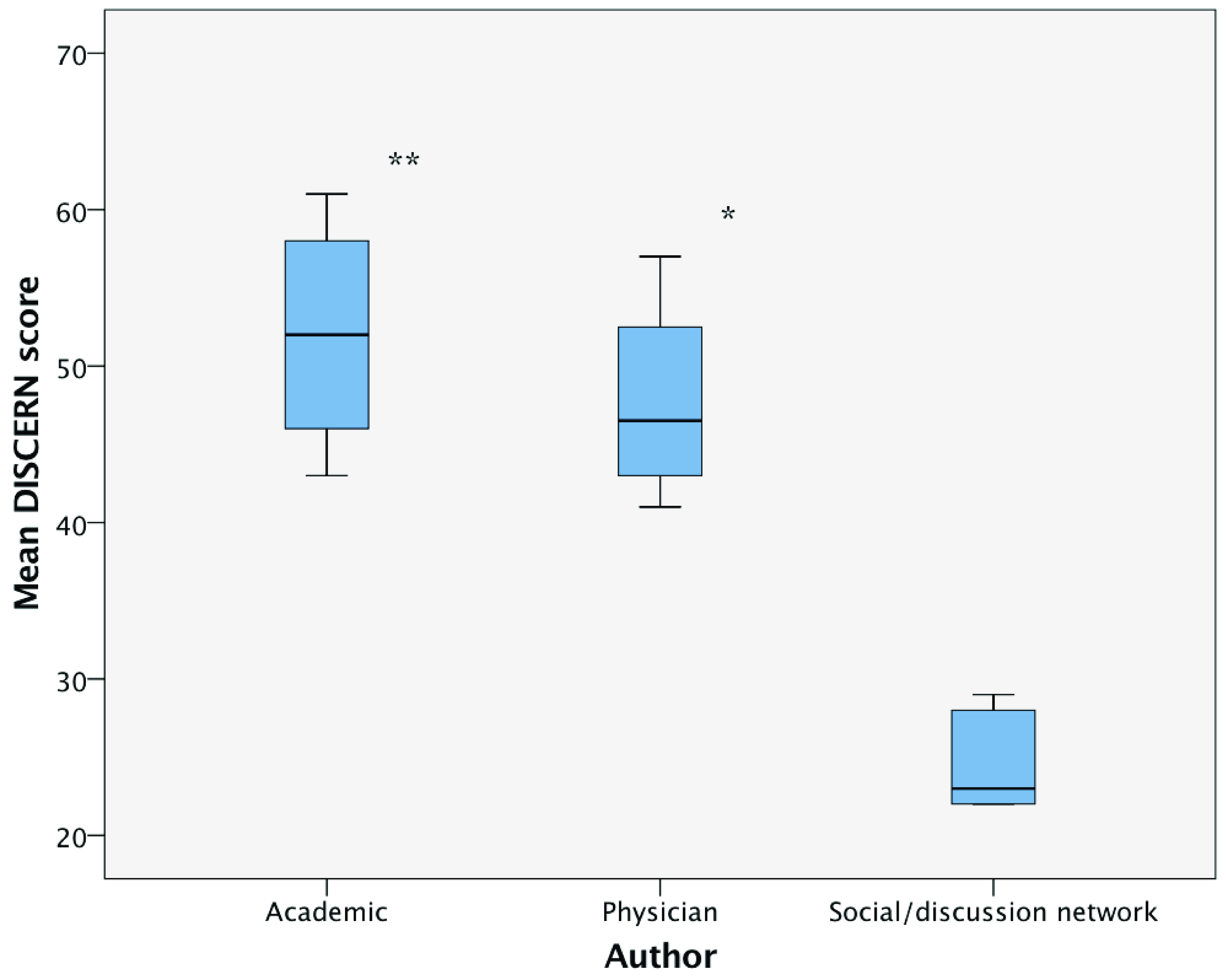
Mean DISCERN scores. Significantly higher for academic ( *P = 0.005*) and physician-related ( *P = 0.032)* websites versus the mean DISCERN score for social/discussion networks.

The DISCERN instrument was used to assess each website. Overall, the mean DISCERN score was low, 40.3 ± 13 out of a maximum of 80. Three of the top five scoring websites were of academic authorship with one being of physician authorship and one Government/NPO. Eight websites (25%) scored 51 or above, representing good quality, with academic authorship associated with both the single highest DISCERN score (61) and the highest mean DISCERN score across all groups (49.7). Six websites (18.75%) scored less than 26 points, representing very poor quality with extensive shortcomings. Average DISCERN scores by authorship groups are shown in Figure 4. Neither HONcode nor the presence of the Information Standard quality mark was associated with a higher mean DISCERN score ( *P=0.08* and *P=0.7*, respectively). Academic and physician-related websites achieved significantly higher mean DISCERN scores than social/discussion networks ( *P = 0.005* and *P = 0.032*, respectively; Figure 5).

## Discussion

There can be no doubt that the Internet has changed the manner in which patients access information. Traditionally, information was passed from doctor to patient in a single direction and decisions were made under the paternalistic guidance of the doctor. In the Internet era, in which information is immediately available, this flow of information is no longer appropriate, nor is it acceptable to patients. More and more, patients are accessing this medical information online and using it to make decisions regarding their own healthcare ^8, 18^. However, the reliability and suitability of these online patient education materials is increasingly being called into question ^8, 13, 14, 16– 18^. The majority of Internet users start their search with a search engine, and most do not trawl beyond the websites from the first three pages returned ^22^. The aim of this study was to assess both the quality and the readability of Internet information relating to anaesthesia for total hip replacement and total knee replacement, using three validated tools to assess readability (FRE, GFI, FKGL) and the DISCERN instrument to assess quality of information obtained. We also looked for websites that displayed HONcode certification or had received the Information Standard NHS quality mark.

Our results show that 81.2% of the websites assessed were above the recommended sixth-grade readability level for PEM. This should be a concern for healthcare providers; many patients will have a limited understanding of the health information available to them online, and thus even those patient education materials that may be of good quality may not be understood. This could have an adverse effect on the informed consent and decision-making process. These findings echo multiple studies over the last decade, suggesting that producing information at an appropriate readability level is still a challenge for healthcare providers ^10, 18, 19, 21^. Encouragingly, our study found that websites that had been awarded the Information Standard NHS quality mark were statistically significantly more likely to achieve the appropriate readability level, suggesting that these are the websites that healthcare providers should be recommending to our patients.

The quality of the websites was assessed using the DISCERN instrument. It is important to note that the DISCERN instrument does not take into account the readability of the material, and thus when recommending websites to patients, healthcare providers should seek websites which are of both high quality and appropriate readability. In our study, only a small number of the websites analysed (25%) scored highly using the DISCERN instrument, which indicates that most PEM related to anaesthesia for TKR and THR available on the Internet are of poor quality. Websites were more likely to achieve high DISCERN scores if the authors were physicians, affiliated with academic institutions or government agencies. Again, this highlights the importance of healthcare professionals directing patients towards more reliable and appropriate PEM.

One of the most significant and disappointing findings from our study relates to the presence or absence of the HONcode seal on websites. Although the HONcode seal indicates that a website has met certain quality and reliability standards, our study did not find that HONcode certified websites achieved higher readability or quality standards than those without the HONcode seal. The Information Standard quality mark was introduced to help patients make informed choices about their condition and options for treatment. In our study, we found that although there was a statistically significant relationship between the presence of the Information Standard quality mark and appropriate reading levels, there was no correlation between this quality mark and quality of information using the DISCERN instrument.

A number of limitations to this study are recognised. We performed our online search in one country, and only analysed PEM from websites written in the English language. The search was limited to the first three pages of returned websites, as it has been shown previously that the general public usually don’t pursue beyond this ^22^. We acknowledge that comprehension of healthcare information is not solely related to readability of text and that other factors, i.e. videos and visual tools, can contribute greatly to a patient’s understanding. The examination of such materials was beyond the scope of this study and previous studies have also acknowledged this limitation ^8, 16^. While readability indices have been validated in the literature, there is no general consensus on which index is best and each one uses a different formula to calculate readability. Scores by different indices may vary substantially. It should also be noted that although there is a large volume of material available to guide users when appraising websites using the DISCERN instrument, there is still the potential for variability among raters, which is a limitation not present when assessing readability.

In conclusion, we aimed to assess the quality and readability of information available online regarding anaesthesia for total knee replacement and total hip replacement. Overall, we found that most of the websites were of poor quality and many had reading levels which were too high for the target audience. These findings echo many other studies that examine online information relating to healthcare ^8, 11, 13, 16, 17, 19, 20^. We found that while the Information Standard NHS quality mark was associated with improved readability, neither the quality mark nor the HONcode were found to have a statistically significant correlation with quality of material. Based on this study we would encourage doctors and other healthcare professionals to be judicious in the websites they recommend to patients, and to consider both the readability and the quality of the information provided.

## Data availability

### Underlying data

Figshare. WorkbookFinalPEM.xlsx. Data figures relating to an internet-based readability and quality control study using recognised quality scoring systems to assess the patient information available online for anaesthesia for total hip and knee replacement surgery. https://doi.org/10.6084/m9.figshare.7940753.v1 ^26^.

Data are available under the terms of the Creative Commons Attribution 4.0 International license (CC-BY 4.0).
