# Supplementary material for: Anaesthesia for total hip and knee replacement: A review of patient education materials available online
Source: F1000Res. 2019 Apr 9;8:416. [Version 1] doi: 10.12688/f1000research.18675.1 (PMC7722535; doi:10.12688/f1000research.18675.1)
Supplement: Peer review history

# Peer review: Reviewer response for version 1

**DOI:** 10.5256/f1000research.20449.r74794

**Published:** 2020-12-07

**Authors:** John McNamara

**Affiliations:** 1 Department of Anaesthesiology, Cork University Hospital, University College Cork, Cork, Ireland

Thank you for the opportunity to review this interesting paper.

 The use of online health information resources is clearly increasing and will continue to do so.  This point is well made by the authors, and appropriate references are well cited.

 This research is timely, in an era when we are increasingly aware of the potential for spread of misinformation online. The medical profession has long been aware of the potential pitfalls of misguided internet searches for health information. It is interesting to determine the prominence of low quality information. The approach of using multiple internet search engines is pragmatic and makes the results of the article applicable. Perhaps inclusion of the term “anaesthesia”, as well as “anaesthetic”, in the search terms would have captured additional relevant websites.

 Overall the article is well-written and easy to read, and describes a topical issue which is not frequently addressed by the medical profession, despite the potential for positive impact on the health of our patients.

Is the work clearly and accurately presented and does it cite the current literature?

Yes

If applicable, is the statistical analysis and its interpretation appropriate?

Yes

Are all the source data underlying the results available to ensure full reproducibility?

Yes

Is the study design appropriate and is the work technically sound?

Yes

Are the conclusions drawn adequately supported by the results?

Yes

Are sufficient details of methods and analysis provided to allow replication by others?

Yes

Reviewer Expertise:

Regional anaesthesia, Cardiac anaesthesia

I confirm that I have read this submission and believe that I have an appropriate level of expertise to confirm that it is of an acceptable scientific standard.

# Peer review: Reviewer response for version 1

**DOI:** 10.5256/f1000research.20449.r58795

**Published:** 2020-01-31

**Authors:** Thomas W. Wainwright, Louise Burgess

**Affiliations:** 1 Orthopaedic Research Institute, Bournemouth University, Bournemouth, UK

Thank you for the opportunity to review this well-written analysis of online patient education materials on anaesthesia for total hip and knee replacement.

 We commend the authors for investigating this relatively under-researched but important topic.

 Overall, they have presented a valuable addition to the literature-base. We do, however have the following suggestions which we believe would strengthen the contribution of this article to healthcare professionals looking to create or direct patients to relevant educational materials online.

 1. Whilst DISCERN is an appropriate tool for assessing the quality of written information about treatment choices, we invite the authors to elaborate on the following points:

- Questions 1-8 of the DISCERN tool addresses the reliability of the publication, and questions 9 to 15 focus on the specific details on the information on treatment choices. The final component of the DISCERN tool is an overall quality score. It may be valuable to the reader for the authors to present the results from each criterion of the DISCERN score (for example, sources of information, aims, relevance, support with shared-decision making etc.), or at least each section (reliability, treatment options, overall quality), so they have specific details on what needs improving in the future design of online patient education materials.
-  Three of the top five scoring websites were of academic authorship. Is it possible for the authors to provide some specific examples from these websites which led to a “high-quality” DISCERN score? Demonstrating examples of exemplar education materials may guide healthcare professionals in the design of high-quality educational materials in the future.
    
- The quality of each website was assessed by two authors using the DISCERN instrument. Were there any discrepancies or disagreement between the two authors whilst evaluating the websites with the DISCERN tool? How were these discrepancies resolved? 

2. We commend the authors for conducting three, validated readability tests. The authors found the Information Standard NHS quality mark was associated with improved readability; however along with HONcode were not found to have a significant correlation with quality. We would be interested to know if the “written passages” selected for the readability tests were all taken from the same section of the websites included in this study to reduce variability.

 3. Search engines: Were the searches conducted on browsers with an empty cache? If not – choosing to include the first three pages of results only would likely create an element of selection bias, whereby the websites are ones familiar to the authors. Hence, the sample chosen in this study may not represent the search of a layperson. 

 4. Finally, we must acknowledge that the search for patient education materials was conducted in September 2017. As the content available to patients online is continuously changing, the results of this search have likely been updated, or replaced, with new materials. It may be useful to re-run the search, so that the published results are as relevant as possible to current practice.

Is the work clearly and accurately presented and does it cite the current literature?

Yes

If applicable, is the statistical analysis and its interpretation appropriate?

Yes

Are all the source data underlying the results available to ensure full reproducibility?

Partly

Is the study design appropriate and is the work technically sound?

Yes

Are the conclusions drawn adequately supported by the results?

Yes

Are sufficient details of methods and analysis provided to allow replication by others?

Partly

Reviewer Expertise:

orthopaedic surgery, physiotherapy, rehabilitation, enhanced recovery after surgery, quality improvement

We confirm that we have read this submission and believe that we have an appropriate level of expertise to confirm that it is of an acceptable scientific standard.

# Response

**Published:** 2020-03-01

**Authors:** Mark Johnson

**Affiliations:** Anaesthesiology and Pain Medicine, Fiona Stanley & Fremantle Hospitals, Western Australia., Australia

Thank you Thomas and Louise for your time and effort in reviewing this paper.
